# Integrating educational knowledge: reactivation of prior knowledge during educational learning enhances memory integration

**DOI:** 10.1038/s41539-018-0027-8

**Published:** 2018-06-25

**Authors:** Marlieke Tina Renée van Kesteren, Lydia Krabbendam, Martijn Meeter

**Affiliations:** 1 Section of Education Sciences and LEARN! Research Institute, Vrije Universiteit Amsterdam, Amsterdam, The Netherlands; 2 Section of Clinical and Developmental Neuropsychology, Vrije Universiteit Amsterdam, Amsterdam, The Netherlands

## Abstract

In everyday life and in education, we continuously build and structure our knowledge. Successful knowledge construction is suggested to happen through reactivation of previously learned information during new learning. This reactivation is presumed to lead to integration of old and new memories and strengthen long-term retention. Additionally, congruency with prior knowledge is shown to enhance subsequent memory. However, it is unknown how subjective reactivation and congruency jointly influence learning in an educational context. In two experiments, we investigated this question using an AB-AC inference paradigm where students were asked to first study an AB (word-picture) and then an AC-association (word-description). BC-associations were either congruent or incongruent and were linked by a common, unknown word (A). During AC-learning, participants were instructed to actively reactivate B (the picture) and report their subjective reactivation strength. Participants were first-year university students studying either psychology or family studies and the stimuli consisted of new information from their curricula. We expected that both reactivation and congruency would enhance subsequent associative memory for the inferred BC-association. This was assessed by cueing participants with C (the description) and asking to freely describe the associated picture. Results show a significant enhancement of both B-reactivation and congruency on associative memory scores in both experiments. Additionally, subjective meta-memory measures exhibited the same effect. These outcomes, showing beneficial effects of both reactivation and congruency on memory formation, can be of interest to educational practice, where effectively building knowledge through reactivation is imperative for success.

## Build knowledge through reactivation

How can we best link information that is learned at different periods in time? This question is important in everyday learning situations such as in educational settings. A team led by Marlieke van Kesteren of the Vrije Universiteit Amsterdam in The Netherlands set out to investigate two ways to achieve this. First, they looked at whether relatedness helps to link old with new information, and second they considered the effects of active reactivation of old information while learning new information. Both of these factors were found to benefit memory for the link between the old and the new information. This shows students and teachers that to build knowledge, we need to actively reactivate related old memories while learning new information.

## Introduction

In everyday life and in education, we continuously build and structure knowledge.^1^ To achieve this, we can integrate separately learned instances by inferring an association between them.^2^ This memory integration process is suggested to happen through reactivation of old information^3^ while learning new information and can help build a consistent knowledge network (or schema) in our brain that in turn serves future learning.^4,5^ Understanding more about how prior knowledge and reactivation affect new learning is important in situations where effective knowledge building is key, such as in education.

The presence of prior knowledge has been consistently shown to enhance learning of new, related information in education research,^6^ psychology,^7,8^ and cognitive neuroscience.^4,9^ Prior knowledge structures are suggested to facilitate many stages of memory processing—such as encoding, consolidation, and retrieval—and reduce the interference of competing memories.^10^ Information that is congruent with prior knowledge is therefore generally better remembered. Little is known, however, about the underlying mechanisms through which new memories get assimilated in prior knowledge structures, and how active reactivation of prior knowledge can benefit this process.

In experiments in psychology and neuroscience, memory integration is often assessed using an AB-AC inference paradigm.^2,11^ Here, participants first learn an association between A and B, and this association is later complemented by an association between A and C. Through the common factor A, participants are expected to be able to reactivate B and form an indirect association between B and C, which in turn enhances BC-memory. While this process of reactivation and association is widely suggested^12,13^ and has been linked to neural reactivation patterns during encoding,^14,15^ there is as yet little evidence linking subjective B-reactivation during AC-learning to the inferred BC-memory. An alternative hypothesis would be that the BC-link is inferred only at test.^12^ Therefore, assessing the link between prior knowledge reactivation strength during learning to subsequent memory performance^16,17^ will provide useful information for educational practice.

**Fig. 1:**
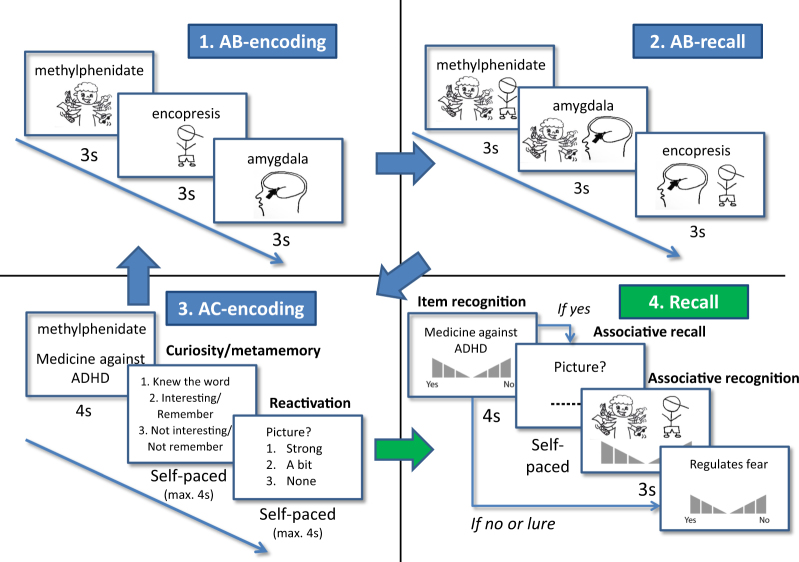
Experimental design. During encoding (blue panels), participants learned 8 ABC-combinations during 10 (experiment 1) or 8 blocks (experiment 2) in three phases: AB-encoding, AB-recall and AC-encoding. In experiment 1, participants were asked whether they thought what they learned was interesting (curiosity), and in experiment 2 they were asked whether they thought they would remember the association (metamemory). In both experiments, they were subsequently asked how strongly they reactivated the associated picture (reactivation). After all blocks were encoded (steps 1–3) and a short math task was performed (not depicted), participants performed a recall task (step 4; green panel) including item recognition (do you recognize this description?), associative recall (given the description, write down the associated picture), and associative recognition (given the description, pick the associated picture) tests respectively. Item recognition and associative recognition tasks also included confidence levels. See for more specific details regarding the design and stimuli the sections “Stimuli” and “Procedure”

Here, we modified the AB-AC inference paradigm to investigate how active reactivation of prior knowledge affects learning. We performed two experiments with two within-subject factors: congruency (i.e. pre-experimental knowledge of a link between B and C) and reactivation (i.e., a subjective score of B-reactivation strength during AC-learning). Stimuli consisted of words (A), pictures (B), and short descriptions (C), all related to topics studied in the Psychology and Family Studies curricula of our university (examples in Fig. 1 and Table 2). During a post-learning memory test, we assessed how these factors affected item recognition for C and associative memory for BC (see Fig. 1).

We expected congruency to enhance subsequent memory, in line with findings that prior knowledge aids retrieval. Moreover, we expected subjective reactivation of the B-picture during AC-learning to also enhance subsequent memory, which would signify a prominent role for both these factors in everyday educational learning. Finally, we also explored potential associations with memory enhancement of other factors in our design: curiosity (experiment 1) and metamemory (experiment 2) judgments after AC-learning.

## Results

For each experiment, we will discuss results in the same order. First, we focus on overall average congruency effects for the AB-test before AC-learning (to show possible initial differences in memory between the conditions) and the memory tests after encoding (item recognition, associative recognition, and associative recall, see Fig. 1), which are also depicted in Table 1. We then move on to investigate the main effect of congruency on these memory tests and its interaction with curiosity/metamemory effects. We finish by investigating reactivation effects on memory, and congruency x reactivation interactions, which are considered the main important analyses of this experiment.

**Table 1:** Overview of paired *t*-test results for congruency tests, experiments 1 and 2

Memory test	Mean congruent (SD)	Mean incongruent (SD)	*T*-value	*P*-value	Effect size (Cohen’s *d*)	95% CI
*Experiment 1*						
AB-recall (%)	96.70 (.04)	96.98 (.05)	.29	.79	−.06	[−.02, .02]
Item recognition (d′)	1.37 (.72)	1.20 (.60)	1.48	.15	.32	[−.07, .40]
Associative recall (%)	60.01 (.21)	41.98 (.22)	4.83	<.001	1.05	[.10, .26]
Associative recognition (%)	98.31^a^ (.05)	90.16 (.10)	4.51	<.001	.98	[.04, .12]
*Experiment 2*						
AB-recall (%)	97.11 (.02)	96.95 (.04)	.21	.83	.04	[−.01, .02]
Item recognition (d′)	1.73 (.70)	1.32 (.65)	2.9	.008	.60	[.12, .73]
Associative recall (%)	62.16 (.13)	40.52 (.18)	7.17	<.001	1.50	[.15, .28]
Associative recognition (%)	97.96 (.03)	93.83 (.07)	2.53	.02	.53	[.01, .08]

*CI* confidence interval

^a^This value was not significantly different from ceiling (100%; (t(20) = −1.65, *P* = .12, *d* = .36, 95% confidence interval (CI) = [−.04, .00]) so this test is not used for further analyses, even though in experiment 2 this value was significantly different from ceiling (t(22) = −3.34, *P* = .003, *d* = .70, 95% confidence interval (CI) = [−.03, −.01])

*P < .05, **P < .001. Degrees of freedom were 20 for experiment 1 and 22 for experiment 2

### Experiment 1

Table 1 shows mean performance as a function of congruency for the AB-test before AC-learning, item recognition, associative recall and associative recognition, and results of *t*-tests comparing congruent and incongruent items (see Fig. 1 and “Procedure” and “Memory tests (recall)” in the Methods section). No differences were found in AB-memory before AC-learning, nor on recognition of the C items. We did find a main effect of congruency on associative recall and associative recognition. However, congruent associative recognition scores were not significantly different from ceiling (t(20) = −1.65, *P* = .12, *d* = .36, 95% confidence interval (CI) = [−.04, .00]), so we decided to not consider this memory task for further analyses. All other measures were significantly different from chance and ceiling.

Subsequently, we tested for combined effects of congruency and curiosity on memory performance. We found no effects on item recognition (congruency: F(1,13) = 1.64, *P* = .22, *η*^2^ = .11; curiosity: *F* < 1; congruency x curiosity: *F* < 1), and only an effect of congruency on associative recall (congruency: F(1,11) = 8.06, *P* = .02, *η*^2^ = .42; curiosity: F(1,11) = 1.90, *P* = .20, *η*^2^ = .15; congruency x curiosity: F(1,11) = 3.0, *P* = .11, *η*^2^ = .21), which is consistent with the main effect of congruency mentioned above. No effects of curiosity on memory performance were found.

**Fig. 2:**
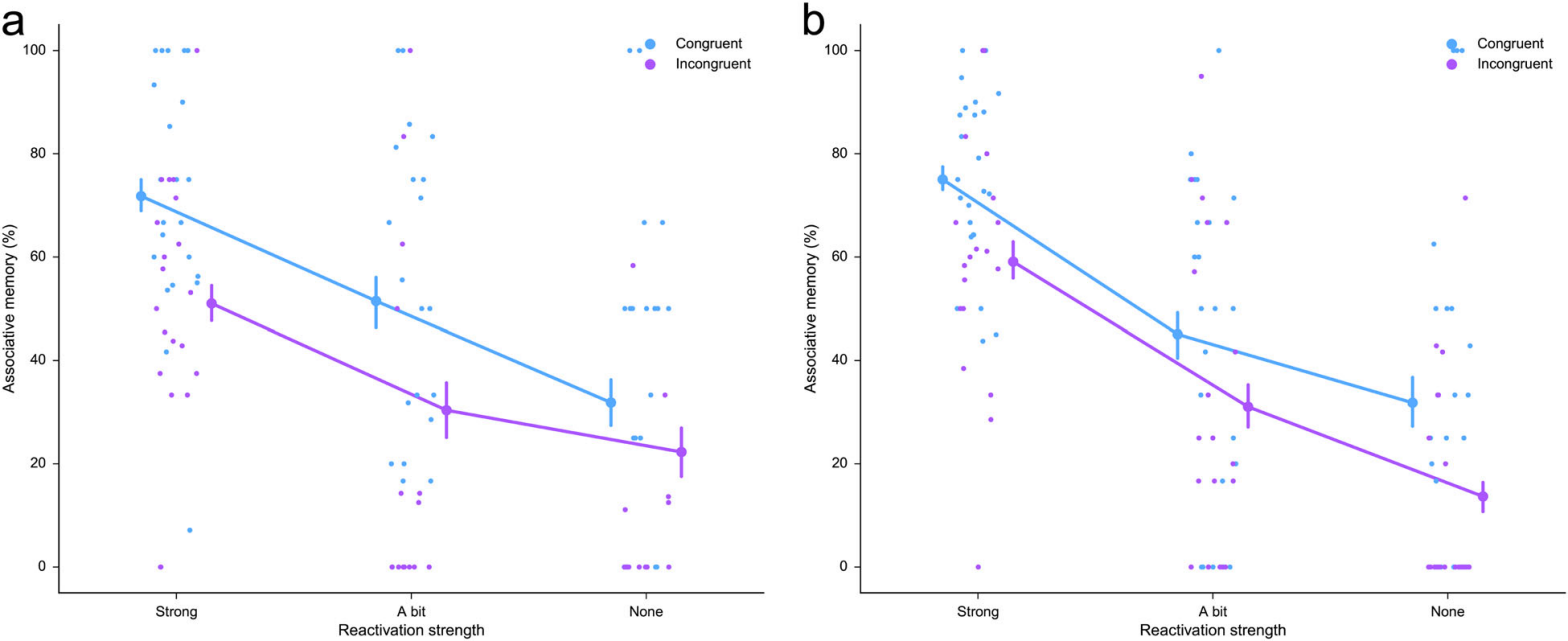
Congruency x reactivation results for experiment 1 (**a**) and experiment 2 (**b**). 2 × 3 Repeated measures ANOVAs showed significant main effects of congruency (*P* = .009 and *P* < .001) and reactivation (*P* = .002 and *P* < .001) on cued associative memory for both experiments. No interactions between congruency and reactivations were found in either experiment. Results shown are group means, as well as all participant means, and error bars indicate Standard Error of the Mean (+/−SEM)

Next, we tested for the combined effects of congruency and reactivation on memory performance (Fig. 2a). We found an effect of reactivation only for item recognition (congruency: *F* < 1; reactivation: F(2,30) = 5.44, *P* = .01, *η*^2^ = .38; congruency x reactivation: *F* < 1) and of congruency and reactivation but no interaction for associative recall (congruency: F(1,12) = 9.87, *P* = .009, *η*^2^ = .45 reactivation: F(2,24) = 8.16, *P* = .002, *η*^2^ = .41; congruency x reactivation: *F* < 1). Congruency and reactivation of the B item thus independently affected associative recall of the indirect BC-association, while only reactivation but not congruency affected item recognition for the C item.

### Experiment 2

For experiment 2 we replaced curiosity ratings with metamemory ratings. Table 1 shows mean performance as a function of congruency for the AB-test before AC-learning, item recognition, associative recall and associative recognition, and results of t-tests comparing congruent and incongruent items (see Fig. 1 and “Procedure” and “Memory tests (recall”) in the Methods section). We found no difference between congruency conditions during the AB-test before AC-learning, showing no differences in AB-memory (see Table 1). We again found significant effects of congruency on associative recall and associative recognition recall, discussed below. Additionally, we now found an effect of congruency on item recognition as well. Moreover, associative recognition scores were significantly different from ceiling in experiment 2 but to remain consistent with experiment 1, and because scores were still very high overall (means 98 and 94% for congruent and incongruent scores respectively), we did not consider this memory task for further analyses.

When testing for combined effects of metamemory and congruency on memory performance, we found main effects of congruency and metamemory but no interaction on both item recognition (congruency: F(1,19) = 5.08, *P* = .04, *η*^2^ = .21; metamemory: F(1,19) = 15.63, *P* = .001, *η*^2^ = .45; congruency x metamemory: *F* < 1) and associative recall (congruency: F(1,17) = 17.73, *P* = .001, *η*^2^ = .51; metamemory: F(1,17) = 19.43, *P* < .001, *η*^2^ = .53; congruency x metamemory: *F* < 1). We subsequently tested combined effects of congruency and reactivation ratings on metamemory ratings to investigate whether participants took congruency and reactivation into account while making their ratings. This analysis showed a main effect of reactivation on metamemory (F(2,32) = 21.09, *P* < .001, *η*^2^ = .57), but no effect of congruency and no interaction between congruency and reactivation (both *F* < 1).

Finally, when testing for combined effects of reactivation and congruency on memory performance (Fig. 2b), we found a main effect of congruency but not of reactivation on item recognition (congruency: F(1,17) = 7.52, *P* = .01, *η*^2^ = .31; reactivation: F(2,34) = 1.64, *P* = .21, *η*^2^ = .09; congruency x reactivation: *F* < 1), and main effects of congruency and reactivation but no interaction on associative recall (congruency: F(1,16) = 21.89, *P* < .001, *η*^2^ = .58; reactivation: Greenhouse-Geisser F(1.36,21.69) = 22.24, *P* < .001, *η*^2^ = .58; congruency x reactivation: *F* < 1). Experiment 2 thus replicates the finding in experiment 1 that both congruency and reactivation enhance associative memory performance for the inferred BC-association.

One issue with the current analyses is the low number of trials in the different reactivation bins. Therefore, we retested the reactivation effects after combining bins “A bit” and “None” together, increasing the amount of trials per bin. Results remained the same: an effect of congruency and reactivation but no interaction. For details about this analysis see Supplementary text S2, where we also report trial count analyses.

## Discussion

In two experiments, we investigated the effects of congruency and subjective reactivation strength on inferring indirectly learned associations. Using an AB-AC inference paradigm with words (A), pictures (B), and descriptions (C), we found that both congruency and subjective reactivation strength enhance associative memory between B and C. This effect was evident in both experiments.

Our results show that pre-experimental congruency between the B and C items strongly affects subsequent memory for the inferred B-C association, and in experiment 2 it also affected recognition of the C-item. These findings fit well with previous literature on schema-related learning,^4,7,18,19^ and extend prior results by showing that congruency effects on memory are also present when congruency (or schema) is only accessible through (active) reactivation.^20^

Additionally, we found that subjective reactivation scores related to subsequent memory such that higher reactivation of B while studying the A–C pairs led to better memory of the indirect B–C associations, both for congruent and for incongruent B–C associations. This reactivation effect was present in both experiments for associative recall, and in Experiment 1 also for recognition of the C-item. The consistent effects of subjective reactivation on associative memory confirm our hypothesis and extend recent findings on recollection of AB-associations before AC-learning^13^ and effects on false memories.^21^ Moreover, our findings can have important consequences for educational practice where active reactivation of previously learned study material can aid subsequent memory.^1,22^

Lastly, we found that curiosity ratings did not relate to subsequent memory, but metamemory ratings did for both item recognition and associative memory. This meant that when participants predicted that they would remember the word (of which we did not test the memory), they were more likely to remember the description, and recall the association between the description and the picture. This suggests that metamemory judgments were at least to some degree accurate, consistent with previous research.^23^ Reactivation ratings were positively related to both metamemory judgments and subsequent memory performance, suggesting that participants based their judgments on the strength of reactivation, which turned out to be a valid indicator of later memory. These findings further inform the debate on the effects of familiarity and accessibility of memories on metamemory.^24^ Moreover, congruency also affected memory, but did not influence metamemory ratings. Participants may thus underestimate the effect of prior knowledge, here operationalized via congruency, on subsequent memory.

As concluded above, these results could be of interest for educational practice. Activating prior knowledge is already emphasized in various forms of teaching.^16,17^ Our results show that reactivation of prior knowledge during learning of new information indeed results in stronger association of new information with existing knowledge networks. This can be contrasted with the practice of considering knowledge as something stored externally, e.g., on the internet, which can be searched on a just-in-time basis.^25^ Our findings provide more reasons for why this “Google effect of memory” is undesirable. Instead, actively reactivating previously learned information when studying new information is beneficial for subsequent memory. Additionally, memory will be stronger when information already has a pre-existing association, such that the reactivated memory is congruent with the newly learned information. However, in cases where information is incongruent with or unrelated to prior knowledge, successful reactivation is still beneficial for subsequent associative memory. These results are in line with several memory theories, such as the schema,^4,8^ retrieval practice,^26^ and reconsolidation theories.^27^ So, our findings stress the necessity for a curriculum where knowledge is slowly expanded and previously learned information is often revisited to allow strong knowledge networks to form.^28,29^

Future studies could thus focus on how this reactivation effect can be successfully implemented in educational situations. One could then think of a replication of the current effect in an educational situation, or a training program where students and teachers are taught to more often or more efficiently reactivate prior knowledge when learning new information. Furthermore, in this experiment we have not tested for memory of the word (A), while in school tests this element is often used as a cue in tests. This is because we were specifically interested in knowledge building through integration between C and the reactivated term B. However, based on previous research,^13–15,30,31^ we expect all associations between A, B, and C to be strengthened as related to subjective reactivation, especially when B is fully recollected.^13^ Nevertheless, a wide range of literature also suggests that AC-memory might be compromised due to B-interference.^11,32^ These accounts require further exploration in future research. Finally, we acknowledge that the reactivation component in our experiment is an extreme simplification of reactivation in classroom learning. However, we propose that the pictures we used represent a more extended knowledge network that, when reactivated, enhances new learning. Finally, neuroimaging studies can reveal how the brain establishes this reactivation effect on memory.^2,33^

In conclusion, our findings in two separate, educationally relevant experiments show that both congruency and subjective reactivation enhance associative memory for inferred associations. Additionally, we find similar effects for metamemory judgments, replicating previous effects of subjective learning on subsequent memory. These findings can be of interest to students and teachers who wish to enhance their retention and understanding of educational subject matter.

## Methods

Two experiments were performed. As only a few features in experiments 1 and 2 were different, we will first describe experiment 1 in detail after which we will note differences for experiment 2. The most important of these differences was that experiment 1 contained a timing manipulation in addition to the congruency manipulation. Here, participants either viewed A for 1.5 s and AC for another 2.5 s, or they viewed AC for the full 4 s. This condition was included because we hypothesized that the former condition would enhance retrieval of B and integration with C. This manipulation did not yield significant effects, so it was dropped for experiment 2. Therefore, we will only describe analyses on the remaining factors (congruency, reactivation, curiosity (experiment 1), and metamemory (experiment 2)) in the main text. More information about the timing manipulation and associated results can be found in Supplementary text S1.

### Experiment 1

#### Participants

In previous studies (for example see ref. ^14,34^), effect sizes of the manipulations used here tended to be large if manipulated within participant. A power analysis assuming large effect size and a statistical power of 0.95 (and standard settings of G-Power^35^) suggests a sample size of at least 23. However, since in such experiments some participants tend to be at floor or at ceiling, we aimed to test at least 30 participants in both experiments. In experiment 1, 31 Participants studying either Psychology or Family Studies at the VU Amsterdam were recruited at the end of their first or second study year. Recruitment was achieved through a university participant system, flyers, and social media. One participant was excluded due to technical issues (coding problem), and nine participants were excluded because they did not have enough stimuli (10 or more in either condition) left for analyses, leaving 21 participants that were included in the analyses (15 from Psychology, five from Family Studies, and one unknown). Of all included participants, age was known for 20 because one participant did not fill out their birth date. The remaining participants were between 18 and 25 years old (mean: 20.33, SD: 1.58), 5 were male, and all self-reported to not be color blind and speak Dutch fluently. For participation, participants provided written informed consent and received either study credit or monetary reward (minimally €10 and maximally €12, depending on time involvement). Ethical approval was obtained before start of the experiments from the ethical committee (VCWE) of the faculty of Behavioral and Movement Sciences of VU Amsterdam.

#### Stimuli

The AB-AC combinations consisted of 80 words (A), clip art type pictures (B) and descriptions (C). For each word, two pictures and two descriptions were created to be able to fully counterbalance congruency (see below). In total, the set thus contains twice as many pictures and descriptions (160) as words, but each participant only encountered 80 of these during encoding. These ABC-combinations were constructed from study material of the studies Psychology and Family Studies of VU Amsterdam, focusing specifically on material learned beyond the first study year. This approach was chosen so participants could build on prior knowledge related to their studies and would be more motivated to study the material.

The ABC-combinations (examples in Fig. 1 and Table 2) were constructed as follows. The words (A-items) were chosen from study material of the Psychology and Family Studies curriculum, second study year and up. All participants, irrespective of their field of study, received the same stimuli. Words were chosen to be not too long (3–37 letters) and never consist of more than two words, so that participants could quickly read them. The set consisted of mostly of Dutch words, but foreign words were chosen when no good Dutch alternative was available (e.g., the English term “Display rules” is used in Dutch pedagogical practices). This resulted in 10 English words, three Latin words, and two abbreviations out of 80.

**Table 2:** Example of an ABC-combination with a word (A), a picture (B) and a description (C)

Word (A)	Picture 1 (B)	Picture 2 (B)	Description 1 (C)	Description 2 (C)
Methylphenidate			Medicine against ADHD	Can cause nausea

For each participant, a combination of four combinations of picture and description was selected randomly such that picture or description salience or memorability could not affect subsequent memory performance. In this case, participants either learned a congruent combination (Methylphenidate—Picture 1—Description 1, or Methylphenidate—Picture 2—Description 2) or an incongruent combination (Methylphenidate—Picture 1—Description 2, or Methylphenidate—Picture 2—Description 1). During item recognition, participants would encounter both the learned and not-learned description, which served as lures. Please note that these pictures are redrawn due to copyright issues. The used pictures were colored clip-art pictures that are available from the authors upon request.

B-items were clip-art pictures selected to have clear lines and colors and to not be aversive in any way. They were obtained through the internet and contained no text unless absolutely necessary for the description (e.g., in case of the Stroop task) and they were judged by the experimenters to be easily describable in a few words. Picture size was set to a maximum of 350 × 350 pixels, with either the height or the width minimally equal to this value to keep the natural dimensions of the pictures (e.g. a landscape-oriented picture would be 350 × 300 pixels whereas a portrait-oriented picture would be 300 × 350 pixels). All pictures were checked manually for clear resolution and size. C-items were descriptions, consisting of maximally five words (range 1–5) and maximally 37 letters (range 5–37). All these descriptions were in Dutch. The BC-associations were chosen such that they could be combined into two congruent and two incongruent associations (see Table 2). For example, for the word “Methylphenidate” a picture of a boy juggling a lot of tasks was coupled with the description “Medicine against ADHD”. Additionally, we added a picture of a sick smiley, coupled with the description “Can cause nausea”. We thus made sure that for the congruent associations, the description always related to something in the picture. The incongruent associations were constructed by combining the picture with the “wrong” description (e.g. boy with “Can cause nausea”, and smiley with “Medicine against ADHD”). We manually checked that each incongruent association did not contain clear possible congruent relations. This resulted in four different possible associations per word that were counterbalanced randomly over participants so individual pairings would not influence average performance. This meant that each participant only learned a random half of the stimulus set, which allowed us to use the other descriptions as lures during the item recognition task (see Recall).

#### Procedure

The experimental procedure consisted of three parts: encoding, math task, and memory tests (recall). Participants were tested individually on a computer in a room shielded from outside noise. The task was presented with Presentation 19.0 (Neurobehavioral Systems) and participants used the computer keyboard to give responses. After giving informed consent and reading task instructions from a sheet of paper, participants performed a practice session consisting of three practice items that were not included in the experiment. After the practice session, they were allowed to ask questions and were given the opportunity to take the practice session again.

#### Encoding

During encoding, an AB-AC inference paradigm was used (Fig. 1) consisting of three items: a word (A), clip-art pictures (B), and a description (C). Participants learned these associations in 10 blocks with 8 associations each, leading to a total of 80 associations. Encoding blocks contained three phases: AB-encoding, AB-recall, and AC-encoding (see Fig. 1). The order of associations within each phase was pre-randomized for each participant separately such that (1) the congruency factor was randomly assigned, (2) each block contained four congruent and four incongruent associations, and (3) order was pseudo-randomized such that no more than three of each factor (congruent or incongruent) followed each other. The stimulus order during AC-encoding was the same as AB-encoding, but AB-recall had a different pseudo-random order with the same boundary conditions. In order to understand what was expected of them, participants were cued with the block number before each block (“Block n”) and with a description of the phase that would follow before each phase (i.e., “Learning”, “Test” etc.). Here, they were also instructed which buttons they had to use if they needed to answer questions (buttons “1” and “2” for AB-recall and buttons “1”, “2”, and “3” for AC-encoding, see below). Each of these cues lasted two seconds. Background color was set to white throughout and the experiment was run on full screen.

First, during the AB-encoding phase, participants encoded eight AB-associations (word-picture), which were depicted in the middle of the screen, word above picture (see Fig. 1). Word color was set to black and word size to 30. Words and pictures were shown for three seconds each with an inter-trial interval (ITI) of one second. Participants were asked to passively look at the associations and try to remember them. After they learned eight AB-associations, participants were tested on each of these associations (AB-recall). They were shown each word again, in a different random order than during AB-learning. This time the word was shown with two pictures underneath, one of which was the correct associated picture and the other was one of the other, randomly selected, pictures they just learned in the preceding AB-encoding phase. The side (left or right) of the correct picture was randomly assigned as well. Participants were instructed to press either “1” for the left or “2” for the right picture on the computer keyboard. After they pressed, they immediately proceeded to the next picture or, if they did not press, the program would continue after three seconds maximum.

Then, participants proceeded to the AC-encoding phase. Here, they were shown each of the eight words again (in the same random order as during AB-encoding), but this time with an associated description, for four seconds (or 1.5 s A and 2.5 s AC, see Supplementary Text S1). Words were again shown in black above the descriptions, which were depicted in blue, both with the same text size. Participants were instructed to again study these associations and additionally try to reactivate the associated B-picture by generating a mental image. After stimulus presentation, participants were asked two questions. First, they were asked to indicate whether they (1) already knew the word, (2) thought the word was interesting, (3) thought the word was not interesting (curiosity). After they answered this question or, if they did not press, after four seconds, they moved on to the second question. Here, they were asked to indicate whether they managed to reactivate the B-picture: (1) strongly, (2) a bit, or (3) not. This trial was again presented for maximally four seconds. For both these questions, participants were instructed to answer with the buttons “1”, “2”, and “3” and answer as quickly as possible to avoid exceeding the answer period. After finishing this phase, they saw a block cue and moved on to the next block in which they learned 8 new associations. Encoding lasted approximately 25 min.

#### Math task

After encoding, participants performed a short math distraction task. Participants were asked to count backwards from a given number with a given step amount (e.g., from 89, count back in steps of 6). After 10 s, they were asked to type in the number they ended with and press “Enter”. Participants followed this procedure for 6 numbers, which lasted a few minutes, depending on how quickly participants answered. This task was solely included to disrupt possible ongoing working memory processes so logfiles were checked to see whether participants performed the task but data was not analyzed.

#### Memory tests (recall)

Following the math task, participants were tested on the learned C-items, first through an item recognition test, then a cued recall test, and finally an associative recognition test for each of the ABC-combinations that they learned during encoding (see recall panel in Fig. 1). Order was pseudo-randomized for each participant such that no more than three items of each condition (congruent and incongruent) followed each other. Lures consisted of all the descriptions that were not learned during encoding (80) and were interspersed pseudo-randomly using the same constraint. Before starting the memory test, participants received a practice trial containing five items of which three were previously seen during the encoding practice round. If they wished, they could do this practice task a second time.

First, participants performed an item recognition task for the C items. They were shown a description in the middle of the screen and were asked to indicate whether they learned this description during encoding. They did this by using a 6-point likert scale ranging from “1” (“very sure I learned this”) to ”6” (“very sure I did not learn this”). If participants indicated they learned an encoded stimulus (i.e., by using buttons 1–3), they continued to the next two memory tasks. Else, they were shown the next description. This trial lasted for four seconds regardless of when participants pressed a button. After answering, participants were shown their answer (with a red marking) on the screen and the screen froze for the remainder of the trial before moving to the next trial.

The second memory task (referred to as associative recall) was a cued recall test of the indirect BC-association where the description (part C) was used as a cue and participants were instructed to answer by typing in a description of the indirectly associated picture (B). Answer time was self-paced, and participants proceeded to the next test by pressing “Enter”. If they did not know the associated picture, they were instructed to press “Enter” immediately. Then, participants received a cue to put their fingers back on the buttons 1–6 and press “1” to continue. This was included to make sure participants could answer quickly in the next test.

That third memory task was an associative recognition test of the BC-association where the description (C) was used as a cue, and participants could choose from two pictures, one of which was the correct answer (B) and the other was randomly chosen from the full set of learned pictures while making sure that this lure picture only appeared as a lure once. The pictures appeared in random order next to each other underneath the description. To answer, participants used the same scale as during item recognition, where buttons 1–3 corresponded to the left picture and 4–6 to the right picture. This trial lasted for three seconds regardless of when participant pressed a button. After answering, participants were shown their answer (with a red marking) on the screen and the screen froze for the remainder of the trial before moving to the next trial.

Time spent on the memory tests depended on performance, specifically because the cued recall task involved typing in the answer in a self-paced manner (see below). Nevertheless, total participation time never exceeded 1.5 h. After finishing the recall task, participants filled out a study-specific questionnaire describing their experiences with the experiment, and a payment form in case they wanted a monetary compensation. They also received a debriefing that stated the goal of the experiment and provided the opportunity to learn about future outcomes of the experiment, and they were invited to ask questions about the nature of the experiment.

#### Analyses and code availability

Analyses were performed using custom Matlab 2015b (The Mathworks) scripts, IBM SPSS Statistics 23, GPower 3.1,^35^ and graphs were created using Jupyter Notebook (http://jupyter.org/) together with the Seaborn package (https://seaborn.pydata.org). All scripts can be found on https://github.com/marliekevk/Integrating-Educational-Knowledge. Stimuli of which the word was indicated to be known (to ensure new learning was going on), or were not responded to in time during AC-encoding were excluded from analyses. Additionally, trials that were not responded to in time during recall were omitted as well. As indicated above (in the Participants section), only participants that still had 10 or more trials left per condition (item recognition hits) after these omissions were included in the analyses (21 participants). For the curiosity and reactivation analyses, the trials that did not include a curiosity or reactivation score were also omitted.

To investigate congruency effects, we used paired-sample two-tailed *T*-tests on performance scores for all three memory tests (d-prime for item recognition, and percentage correct for associative recall and recognition), and one-sample two-tailed *T*-tests to detect differences from chance performance. D-prime was calculated by *Z*-scoring the hits and false alarms scores and calculating the difference between these measures (Z(hits)—Z(FA)).^36^ Confidence level was not further considered because of low trial numbers. In cases where there was a 0% false alarm rate in a certain condition, false alarms were recalculated using the MacMillan method^37^ (false alarm rate = 0.5/*n* where *n* is the maximum number of trials per condition). Correctness of the answers in the associative recall test (where participants were instructed to freely type in their answer) was assessed by hand by two independent raters that rated each description with either 0 (incorrect), 0.5 (partly correct) or 1 (correct). After independent rating, the two raters reached consensus on the items where they disagreed to come to a final rating per item. Performance in the final associative recognition test was calculated as the proportion of hits, independent of confidence.

To investigate effects of curiosity and reactivation, we calculated average memory scores for each bin (2 scores for curiosity and 3 scores for reactivation, see Encoding for details on these ratings). We subsequently assessed effects of within-subject factors congruency and curiosity (2 × 2) or reactivation (2 × 3) using a repeated-measures ANOVA. Note that because trials were not equally divided over curiosity, metamemory (see below Exp. 2), and reactivation factors, statistics for these factors were not necessarily equal in the different ANOVAs. For that reason, we report first paired-sample *T*-tests for main effects as well (see above), which would normally be redundant. Alpha was set at .05 throughout.

### Experiment 2

For experiment 2 we made a few adjustments to the experimental design. Only the differences are detailed below, the rest of the procedure and design was the same as in experiment 1.

#### Participants

Thirty participants were recruited at the end of their first study year (one year after experiment 1). One participant was excluded due to technical issues (no encoding logfiles were stored) and six participants were excluded because they did not have enough stimuli (ten or more in either condition) left for analyses, leaving 23 participants that were included in the analyses (16 from Psychology, five from family studies, and two unknown; seven male; mean age 20.32 SD 1.56, range 18–24).

#### Stimuli

We omitted the 16 associations that participants in experiment 1 indicated were most familiar from the set, leaving 64 associations divided over 8 blocks of 8 associations. These consisted, next to Dutch words, of seven English and two Latin words.

#### Procedure

The timing condition was omitted because we did not find effects in experiment 1 (see SI), leaving only the congruency condition. Because of a null-effect for curiosity ratings as well, we now asked participants to indicate metamemory judgments (“how well do you think you will remember the word”) after AC-learning instead of curiosity ratings. More specifically, participants were asked whether they (1) already “knew”, (2) thought they were “going to remember” or (3) thought they were “not going to remember” the word. The rest of the procedure was the same, and for randomization only the congruency factor was taken into account. Because of the decrease in number of associations, this experiment was a bit shorter than experiment 1: approximately 20 min for encoding and 1 h and 20 min in total.

### Data availability

Data are openly available on Harvard Dataverse: 10.7910/DVN/UQV9H4. Supplementary information (SI) is available online, and all original stimulus combinations are available upon request because of copyright issues.

## Electronic supplementary material

Supplemental material

## Electronic supplementary material

**Supplementary information** accompanies the paper on the *npj Science of Learning* website (10.1038/s41539-018-0027-8).
