# Supplementary material for: Integrating educational knowledge: reactivation of prior knowledge during educational learning enhances memory integration
Source: NPJ Sci Learn. 2018 Jun 25;3:11. doi: 10.1038/s41539-018-0027-8 (PMC6220240; doi:10.1038/s41539-018-0027-8)
Supplement: Supplementary file 1 — Supplemental material [file 41539_2018_27_MOESM1_ESM.docx]

**Supplementary Information for van Kesteren et al. “Integrating educational knowledge”**

**Supplementary text S1**

**Timing manipulation (experiment 1)**

In experiment 1, we also added a timing manipulation next to the congruency manipulation. We here hypothesized that sequential timing, priming A before showing C (timing1), would benefit BC associative memory as opposed to when A and C are presented jointly (timing2), because it would enforce participants to reactivate B more strongly. However, this manipulation did not yield any significant effects on subsequent memory, so we omitted it from Experiment 2 and decided to not explain it in the main text due to space restrictions. Below, we describe this timing manipulation in Experiment 1 and the associated results.

***Participants***

For the timing analyses, one participant was different from the congruency analyses (i.e. excluded for congruency but included for timing and vice versa). 21 Participants were included which were all between 18 and 25 years old (mean: 20.40, SD: 1.54). For the rest of the participant information see the main text.

***Methods***

Within blocks, the timing conditions were arranged in a similar way as the congruency condition. There were four items for each factor (timing1 or timing2) and items were randomized such that no more three of each factor (timing1 or timing2) followed each other. During AC-learning, A and C were either presented with a timing delay (i.e. A was presented for 1.5 seconds alone and then C was presented together with A for 2.5 additional seconds; timing1) or at the same time (i.e. A and C were on the screen for the full four seconds; timing2). Using the trial constraints mentioned in the main text left us with only 3 participants that could be included in the congruency x timing analyses. Therefore, we decided to analyze the congruency and timing conditions separately. Below are the analyses for the timing condition, the analyses for the congruency condition are in the main text.

***Results***

For the timing manipulation, there were no effects of timing on performance on any memory measure (see Table 3). When testing effects of timing and curiosity on memory performance, we found a timing and timing x curiosity effect on item recognition (timing: F(1,11) = 6.96, p = .02, η^2^=.35; curiosity: F(1,11) = 1.68, p = .22, η^2^ = .11; timing x curiosity: F(1,11) = 20.38, p = .001, η^2^ = .61) and no effects on associative recall (timing: F(1,13) = .50, p = .50, η^2^ =.04; curiosity: F(1,13) = 1.11, p = .32, η^2^ = .09; timing x curiosity: F(1,130) = 1.54, p = .24, η^2^ = .12). When testing effects on timing and reactivation on memory performance, we found a reactivation effect for item recognition (timing: F(1,15) = .67, p = .43, η^2^ =.04; reactivation: F(2,30) = 5.63, p = .008, η^2^ = .27; timing x reactivation: F(2,30) = .58, p = .57, η^2^ = .04) and for associative recall (timing: F(1,12) = .28, p = .60, η^2^ =.02; reactivation: F(2,24) = 13.67, p < .001, η^2^ = .53; timing x reactivation: F(2,24) = 3.09, p = .06, η^2^ = .21), but no timing effects. Within the timing manipulation we thus found no effects of reactivation on any measure of subsequent memory performance and a small effect of timing on item recognition and curiosity. These results possibly indicate that consecutive presentation of A and C slightly boosts item memory for C specifically but since we did not detect this effect in the paired-samples T-test directly contrasting D’ measures of item recognition, and there is no such effect for associative memory either, this finding should be interpreted with caution.

**Table S1**

Overview of paired t-test results for timing tests, experiment 1.

| **Memory test** | **Mean timing 1** | **Mean**  **timing 2** | **T-value** | **P-value** | **Power (Cohen’s d)** | **95% CI** |
| --- | --- | --- | --- | --- | --- | --- |
| Item recognition (d’) | 1.30 (.67) | 1.16 (.53) | 1.21 | .24 | .26 | [-.10, .39] |
| Associative recall (%) | 49.11 (.21) | 52.91 (.20) | -1.20 | .25 | -.26 | [-.10, .03] |
| Associative recognition (%) | 94.84 (.07) | 94.72 (.08) | .07 | .95 | .01 | [-.03, .04] |

Note: Df = 20, CI = Confidence Interval

**Supplementary text S2**

**Extra tests of reactivation effects**

1. Congruency x reactivation (2x2) effects for experiments 1 and 2 when combining the values of bins 2 and 3 together, contrasting strong reactivation to little or no reactivation.

Experiment 1

- Item recognition: congruency F<1; reactivation F(1,20) =11.89, p = .003, η^2^ = .37; congruency x reactivation F<1.
- Associative recall: congruency F(1,18) =10.5, p = .005, η^2^ = .37; reactivation F(1,18) =33.5, p < .001, η^2^ = .65; congruency x reactivation F<1.

Experiment 2

- Item recognition: congruency F(1,19) = 13.50, p = .002, η^2^ = .42; reactivation F(1,19) =5.58, p = .03, η^2^ = .23; congruency x reactivation F(1,19) = 1.08, p = .31, η^2^ = .05.
- Associative recall: congruency F(1,19) =28.99, p <.001, η^2^ = .60; reactivation F(1,19) =31.47, p < .001, η^2^ = .62; congruency x reactivation F<1.

1. Trial count congruency x reactivation (2x3) effects for experiments 1 and 2.

Experiment 1

- Item recognition: congruency F<1; reactivation F(2,40) = 13.57, p <.001, η^2^ = .40; congruency x reactivation F(2,40) = 4.86, p = .009, η^2^ = .20.
- Associative recall: congruency F<1; reactivation F(2,40) = 17.84, p < .001, η^2^ = .47; congruency x reactivation F(2,40) = 7.02, p = .002, η^2^ = .26.

Experiment 2

- Item recognition: congruency F(1,22) = .26, p = .62, η^2^ = .01; reactivation: Greenhouse-Geisser F(1.35,29.65) =7.04, p = .008, η^2^ = .24; congruency x reactivation F(2,44) = 15.10, p < .001, η^2^ = .41.
- Associative recall: congruency F(1,22) = 10.17, p =.004, η^2^ = .32; reactivation: Greenhouse-Geisser F(1.28,28.09) = 10.09, p = .002, η^2^ = .31; congruency x reactivation: Greenhouse-Geisser F(1.49,32.73) = 17.76, p < .001, η^2^ = .45.
